# A study on the positive and negative effects of different supervisor monitoring in remote workplaces

**DOI:** 10.3389/fpsyg.2024.1383207

**Published:** 2024-04-18

**Authors:** Shuang Li, Yumei Wang

**Affiliations:** ^1^College of Economics and Management, Mianyang Teachers’ college, Mianyang, China; ^2^School of Business Administration, Southwestern University of Finance and Economics, Chengdu, Sichuan, China

**Keywords:** remote work, interactional monitoring, electronic monitoring, work engagement, deviant behavior, self-efficacy, job demand-resource(JD-R) model

## Abstract

The current academic research on whether and how the different supervisor monitoring effect in remote workplace is relatively scarce. Based on the Job demand-resource (JD-R) Model, this study proposes that as a kind of work resource, interactional monitoring will enhance employees’ self-efficacy, further enhance remote employees’ work engagement and reduce their deviant behaviors. While as a kind of work requirement, electronic monitoring will decrease employees’ self-efficacy, further reduce remote employee’s work engagement and increase their deviant behaviors. This study gets the empirical date of 299 employees who experienced remote work. Amos 23.0, SPSS 23.0 software and process plug-in were used to do the hierarchical regression, bootstrap and simple slope analysis, so that to test the hypothesis. This study broadens the research situation and mechanism of different supervisor monitoring, so as to enrich the comprehensive understanding of the effect of them, and also to provide some inspiration and reference for relevant management practices.

## Introduction

Remote work was proposed in the 1970s (Golden and Eddleston, 2020), and the global remote work practice was intensified by COVID-19 in 2020 (O’Brien and Yazdani Aliabadi, 2020). More than 3 years since the outbreak of COVID-19, the proportion of remote work has significantly increased in Europe, America, and Asia (Huo et al., 2022). Data shows that in May 2020, more than 65% of people in the United States worked remotely at home (Gallup, 2020). In China, according to the “China Remote Work at Home Development Report” released by Zhaopin and Beijing National Development Research Institute in 2022 (referred to as the development report), the number of remote work at home job postings in 2021 after COVID-19 was 3–5 times than before it. The development report also predicts that remote work has shown advantages and prospects independent of the impact of COVID-19 from the perspectives of the macroeconomy, enterprises, employees, and countries and governments. Under this background, it is necessary to pay attention to the impact of remote work on enterprises and employees.

The most crucial feature of remote work is spatial isolation from the organization (Xiao, 2019), resulting in invisibility between superiors and subordinates. Therefore, superiors will worry about whether remote employees are working hard. Previous studies have found that in remote workplaces, many employees have difficulty maintaining focus due to the lack of direct supervision from superiors (Bloom et al., 2015), which may reduce work efficiency (Leslie et al., 2012). Therefore, due to concerns about the work status of remote employees, even if they cannot meet face-to-face, leaders will take feasible measures to achieve adequate supervision. The more common ones are electronic supervision (from completely non-interactive camera surveillance and wearable devices with GPS tracking systems to interactive supervision) and interaction supervision, such as regular meetings and informal communication on social platforms (Wu et al., 2020). So, this article focuses on whether and how different supervision methods of superiors produce effects in the remote workplace.

The current research on the effect of supervisors’ monitoring is mainly based on traditional offline workplaces. The study finds that different monitoring methods have different effects, and relevant research mainly explains this from the perspectives of social exchange theory (Liao and Chun, 2016; Son et al., 2017) and self-determination theory (Zhou, 2003; Mishra and Ghosh, 2020). Regarding social exchange theory, Son et al. (2017) treats monitoring as a whole and believe it will destroy the exchange between leaders and members, further hindering employees’ creativity and knowledge-sharing behavior. However, Liao and Chun (2016) distinguish supervisors’ monitoring as interactive and observational. The former can show subordinates that supervisors are willing to listen to their ideas and concerns through personal interactions initiated by supervisors with subordinates, which can positively impact subordinates’ performance. The latter evaluates and observes subordinates without seeking their opinions, and observational monitoring may cause subordinates to lose focus on work tasks and lead to negative attitudes, thus hurting subordinates’ performance. However, empirical research has found that these two monitoring forms are different but not opposite. Regarding self-determination theory, Mishra and Ghosh (2020) found that subordinates who report to supervisors who demonstrate an interactive monitor style may feel that the relationship with their supervisors can meet their basic needs for autonomy, competence, and relatedness, thus enhancing their satisfaction. Conversely, subordinates who report to supervisors using an observational monitor style may not meet their basic psychological needs in the supervisor-subordinate relationship, leading to job dissatisfaction. This study sorts out the characteristics of interactive and electronic monitoring and finds that interactive monitoring can be regarded as support from the organization and supervisors (Mishra and Ghosh, 2020), a work resource (Bedi, 2021), while electronic monitor brings pressure to perform work tasks through requirements, a work demand (Zhou, 2003). Therefore, this study uses the Job Demands-Resources (JD-R) model as a theoretical perspective to explore the differential impact of two remote workplace monitoring forms- interactive and electronic monitoring - on employees’ work engagement and deviant behavior in remote work.

In addition, existing research has demonstrated that self-efficacy can mediate between external environmental factors and individual states (Huang and Chen, 2012). Therefore, when facing the new working environment of remote work, it is worth further exploring whether external monitoring can affect individual employees through self-efficacy as a mediator. Secondly, under the guidance of the Job Demands-Control Model, existing research has also confirmed the differential effects of job demands and job control on self-efficacy (Huang and Chen, 2012). Based on this, this study must explore whether the differential effects of interactive monitoring (work resources) and electronic monitoring (work demand) on self-efficacy exist in remote work. Finally, the most essential reason for introducing self-efficacy is determined by the Job Demands-Resources Model selected in this study. The Job Demands-Resources Model explains job burnout and psychological capital, including self-efficacy dimensions. In summary, this study introduces self-efficacy as an individual resource as a mediator to further explore whether and how the two forms of monitoring in remote workplaces – interactive and electronic – have differential impacts on employee work engagement and deviant behavior.

**Figure 1:**
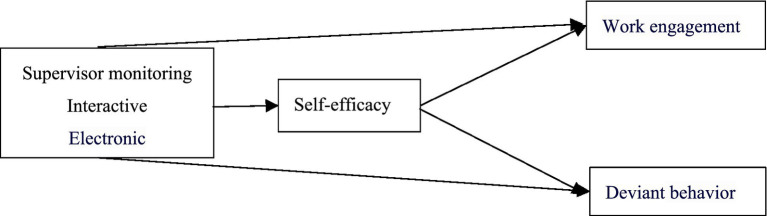
Proposed theoretical model.

This study aims to supplement and contribute to existing relevant research in the following aspects: Firstly, this study enriches the research on leadership and employee behavior in remote workplaces. Secondly, this study extends the research context of supervisor monitoring. Finally, under the guidance of the Job Demands-Resources Model (JD-R), introducing self-efficacy as a mediating mechanism is unique in research perspective. Besides, it further distinguishes the effects of interactive and electronic monitoring from different research contexts and perspectives to enrich the comprehensive understanding of them and their effects. The research results also provide some insights and references for relevant management practices (how to increase work engagement and reduce deviant behavior in remote workplaces). The research model is shown in Figure 1.

## Theory and hypotheses

The academic concept of Supervisor Monitor has a long history, and there are mainly three views: the first is from the perspective of control, which believes that Supervisor Monitor is a way of controlling individual and organizational performance. The second is from the perspective of situational leadership theory, which considers monitoring an essential task of leadership. The third is from the perspective of information, which believes that supervisor monitoring is mainly the behavior of supervisors to collect information about subordinate work progress and effectiveness (Khan et al., 2020). The third concept is the most commonly used, which means that monitoring is about collecting work-related information (Holt et al., 2017). Through information collection, on the one hand, supervisors can ensure that employees follow instructions, perform tasks in the expected way, and refrain from doing anything that supervisors might disapprove of, thus aligning employee behavior with organizational goals (Zhou, 2003; Khan et al., 2020). On the other hand, it is also for performance evaluation (Khan et al., 2020). Therefore, similar concepts include neutral performance monitoring (Performance Monitor) and negative close monitoring (Close monitoring). Based on various characteristics, scholars distinguish monitor types based on supervisors’ methods of collecting subordinate information. Among them, the behavior of collecting subordinate work progress and results in information without direct input from subordinates is called observational monitoring, a top-down monitoring method (Liao and Chun, 2016). In remote work, it is impossible to observe face-to-face directly, but by electronic information technology, so it is also called an electronic monitor (Tomczak et al., 2018). The monitoring method of obtaining information directly from subordinates is interactive monitoring, such as holding meetings with subordinates to understand their expectations, opinions, and feedback on work arrangements and other related issues (Liao and Chun, 2016). Especially, the study considers interactive monitoring during the purely remote work periods; the forms include communicating work matters to collect information in the remote workplaces (such as online meetings, telephone meetings, instant chatting, and so on) but do not include any face-to-face communication. In remote work, the physical isolation of the workplace may cause supervisors to doubt their subordinates’ work performance, so they will try to implement monitoring to evaluate employees’ constructive and destructive behaviors (Ahmed et al., 2022). In this context, supervisors generally use two forms of information collection: electronic and interactive monitors. Whether and how these two monitoring forms affect remote employees’ constructive and destructive behaviors requires theoretical and empirical exploration.

In the traditional face-to-face workplace, research on the role and outcomes of supervisor monitor has yielded inconsistent conclusions, with both positive and negative effects. From a positive perspective, Larson and Callahan (1990) found through experimental research that when task execution is monitored (compared to when it is not monitored), the amount of work completed on the experimental task significantly increases. This is because monitoring increases employees’ perceived importance of tasks, thereby enhancing productivity. Rietzschel and Slijkhuis (2014) found that close monitoring improves employees’ role clarity, positively impacting job satisfaction, work motivation, and job performance. From a negative perspective, numerous studies have suggested that supervisor monitoring decreases employees’ perceived autonomy and leader-member relationships, leading to negative outcomes such as decreased job satisfaction, work motivation and attitude, job performance, creativity, work effort, and knowledge sharing (Niehoff and Moorman, 1993; Rietzschel and Slijkhuis, 2014; Son et al., 2017; Kim, 2020). Furthermore, when classifying interactive and electronic monitors, the positive effects of interactive monitors have generally been consistent in the context of a traditional face-to-face workplace. Liao and Chun (2016) suggest that interactive monitoring is constructive supervision that promotes trust in leadership, enhances leader-member exchange, and fosters positive feedback-seeking behavior, further promoting employee innovation. Wu et al. (2020) found that interactive monitoring enhances employees’ psychological safety, ultimately promoting their trust in management. Khan et al. (2020) studied sales personnel and found that interactive monitoring enhances their work engagement, promoting job performance. Mishra and Ghosh (2020) found that interactive monitoring demonstrates supervisor support for subordinates, promoting job satisfaction. However, there are inconsistent conclusions regarding the role of electronic monitors. On the one hand, some studies have confirmed the positive effects of electronic monitors from different perspectives. For example, electronic monitoring can encourage employees to follow regulations and improve their behavior, preventing counterproductive work behaviors (Pierce et al., 2015; Tomczak et al., 2018). On the other hand, the negative effects of electronic monitors have also received attention. For example, the electronic monitor sends employees the message that they are performing poorly, lack commitment, or are untrustworthy, which in turn leads them to engage in deviant or counterproductive behaviors, reducing entrepreneurial enthusiasm and inhibiting innovation (Holland et al., 2015; Liao and Chun, 2016; Martin et al., 2016; Wu et al., 2021). Holt et al. (2017) studied the perspectives of privacy and ethics and found that electronic monitoring can reduce work acceptability, moral perception, and job satisfaction. In addition, scholars have also found that employees’ perceptions of electronic monitoring can lead to different outcomes. For example, through research, Haley et al. (2012) found that employees’ positive intentions toward electronic monitoring strengthen organizational communication and reduce turnover rates. Conversely, negative views of monitoring weaken communication with the organization and increase turnover rates. Samaranayake and Gamage (2012) studied software industry employees and found that perceived relevance to work and personal judgments of effectiveness are two variables measuring electronic monitor perception. These variables are positively related to job satisfaction, meaning that software employees who are satisfied with their work believe that electronic monitoring is relevant and improves their work quality. However, perceived privacy infringement from electronic monitors is negatively related to job satisfaction. A further review of the literature on the research context of supervisor monitors found very little research on the role and outcomes of supervisor monitors in remote work settings. However, in practice, supervisor monitoring in remote workplaces is quite common. For example, instant messaging tools are commonly used for communication to monitor (some interviewees reported that they feel that supervisors are more frequently tagging people in groups). Therefore, it is necessary to conduct more empirical research to explore whether interactive and electronic monitoring can achieve positive outcomes for employees in remote workplaces while mitigating negative outcomes. In remote workplaces, supervisors’ most intuitive concerns are whether employees work diligently and engage in behaviors that damage organizational interests, such as “gaming the system.” Therefore, this article aims to explore how supervisor interaction and electronic monitoring impact employee work engagement and deviant behavior in remote workplaces within the context of existing literature and management practices.

### Supervisor interaction monitoring and work engagement, deviant behavior in remote workplace

Interactive monitoring is a way to collect information about subordinates’ work by holding regular meetings with them or inviting individual subordinates to participate in discussions (Liao and Chun, 2016). In the context of remote work, as it is not visible like traditional face-to-face work, employees cannot participate in organizational activities and communicate in the typical organizational behavior, leading to a decrease in direct interactive contact with colleagues and managers (Xiao, 2019), which further increases leaders’ concerns about employees’ work conditions. For example, some studies have found that in remote work, lower visibility may cause superiors to perceive and feel that remote workers are “slacking off” (DeRosa et al., 2004). However, with the development of modern communication technology, leaders have become very convenient and frequent in their interactions with remote employees (Barsness et al., 2005). Compared with face-to-face work, organizations and superiors have more constraints and norms on remote employees. For example, keeping connected anytime and anywhere has become a fundamental norm of remote work (Derks et al., 2015), using interactive monitoring to compensate for the decrease in face-to-face supervision norms. Therefore, interactive monitoring is a necessity and feasible in the context of remote work. As in traditional face-to-face workplaces, interactive monitoring in remote workplaces can even more conveniently provide employees with the following opportunities: (i) to understand the expectations and needs of supervisors; (ii) to explain errors or unsatisfactory performance to supervisors and inform them of their achievements that have not been reported or may be overlooked; (iii) to express personal opinions, concerns, and dissatisfaction (Liao and Chun, 2016). Therefore, through interactive monitoring, supervisors can provide subordinates with specific work resources. On the one hand, through communication and feedback, employees can be provided with resources to solve problems. On the other hand, participating in public discussions can convey good intentions, strengthen constructive working relationships with subordinates, and enhance their emotional resources (Tjosvold, 2008). In the context of organizational isolation of remote work, strengthening interactions with subordinates through interactive monitoring can enhance their psychological resources (Xiao, 2019).

Therefore, in the context of long-term remote work (especially when forced to work remotely), how alleviating employee fatigue and distractions, enhancing work engagement, strengthening identification, and making employees more focused, more energetic, and more willing to contribute is a problem that deserves management attention (Hu and Zhang, 2022). Many studies have confirmed that work resources, including emotional and psychological resources, are the prerequisite for enhancing work engagement (Schaufeli and Bakker, 2004; Bakker et al., 2008; Halbesleben et al., 2014; Rahmadani et al., 2020; Liu and Wen, 2022; Zhan et al., 2022; Zheng et al., 2022). Therefore, this study hypothesizes that the interactive monitoring of superiors to increase subordinates’ work resources in remote work can promote employees’ work engagement.

*H1a*: In remote workplaces, supervisor interactive monitoring will increase subordinates’ work engagement.

Workplace deviant behavior is voluntary behavior that violates important organizational norms, either threatening the organization or its members or causing harm to both (Robinson and Bennett, 1995). Its harmful impact on the organization is very concerning, as most employees engage in some level of deviance, causing billions of dollars in productivity and other costs to the organization each year (Mackey et al., 2021). Therefore, it is essential to understand the reasons for deviance and minimize and address it. Previous research has found that in remote workplaces, supervisors cannot intervene in employees’ remote work behavior due to the lack of direct monitoring, which may lead to an increase in employee deviance and a failure to ensure work efficiency (Leslie et al., 2012). Therefore, whether interactive monitoring by supervisors can reduce deviance needs further exploration. Previous research has explained why employees engage in workplace deviance from multiple perspectives, such as emotional event theory (Bordia et al., 2008), stress transaction theory (Mawritz et al., 2014), social learning theory (Mawritz et al., 2012), and personality theory (Meyer et al., 2014). However, scholars have found that these studies have certain consistencies. Furthermore, through meta-analysis, it has been found that the consistencies are reflected in the dominance of social psychology and resource-based theories in exploring the causes of deviance (Mackey et al., 2021). From a resource perspective, it has been found that general work resources hurt employee deviance (Wilson et al., 2015), which means that increasing employees’ work resources can help reduce their deviance. Therefore, this study hypothesizes that interactive monitoring by supervisors in remote workplaces to increase subordinates’ work resources can reduce employee deviance. Therefore, the following hypothesis is proposed:

*H1b*: Supervisor interactive monitoring in remote workplaces will reduce subordinates’ deviant behavior.

### Supervisor electronic monitoring and work engagement, deviant behavior in remote workplace

Electronic monitoring refers to a form of supervision that utilizes modern computer technology to continuously collect data or information on employees, which may involve the use of surveillance cameras, computer, and telephone email monitoring systems, as well as wearable devices or mobile phone applications with global positioning system (GPS) tracking applications (APPs) (such as DingTalk) (Holt et al., 2017). Electronic monitoring occurs without direct input from the subordinate during information collection; it is a typical form of observation monitoring (Tomczak et al., 2018), the only observation monitoring that can be achieved in remote workplaces. Regarding electronic monitoring, researchers believe it is like a work discipline, where the supervised person feels the constant authority of supervision and manages their behavior. Whether electronic monitoring is overt or covert, the mere feeling that an individual may be monitored, even if it does not occur, can be a powerful management tool and potentially have profound implications for individuals (Holland et al., 2015). Therefore, electronic monitoring puts pressure to perform work tasks as required, which is a job demand (Zhou, 2003).

From the perspective of employees’ work engagement, research on the impact of job demands on reducing employees’ work engagement is relatively mature. Specifically, by studying different occupational groups, it has been found that job demands significantly negatively impact work engagement. For example, Liu and Wen (2022) studied primary and secondary school teachers and found that job demands significantly inhibited their work engagement. Chen et al. (2019) studied nurses and found that the higher the job demands of nurses, the less satisfactory their work engagement. Chen and Fellenz (2020) surveyed service industry employees and confirmed that personal job demands reduce employees’ work engagement. Therefore, this study inferred that electronic monitoring would give employees a certain pressure and requirement as an observation supervision form for employees in remote workplaces, thereby reducing remote employees’ work engagement. In summary, this study proposes the following hypothesis:

*H2a*: Supervisor electronic monitoring in remote workplaces will reduce subordinates’ work engagement.

From the perspective of employees’ deviant behavior, previous studies have confirmed that the pressure caused by job demands can lead to deviant behavior (Roberts, 2012; Mawritz et al., 2014; Bazzy and Woehr, 2017). As face-to-face supervision is impossible in remote workplaces, electronic monitoring is mainly limited to the “cyber” level. Therefore, it may reduce employees’ cyber-deviant behavior, but the increased sense of pressure caused by job demands makes employees engage in deviant behavior to recover (Fan et al., 2021). Furthermore, previous studies have found that continuously increasing job demands can impose significant psychological pressure on employees, and employees’ deviant behavior results from the complex interaction of environmental stressors (Cui et al., 2021). Specifically, as electronic monitoring in remote work is a type of pressure that requires tasks to be performed, it can affect employees’ evaluation of environmental cognition (Cui et al., 2021), leading to deviant behavior. Therefore, this study hypothesizes that electronic monitoring will lead to more pronounced traditional deviant behavior. Thus, this study proposes the following hypothesis:

*H2b*: Supervisor electronic monitoring in remote workplaces will increase subordinates’ deviant behavior.

### Mediating role of self-efficacy

Self-efficacy refers to an individual’s belief in their ability to organize and execute specific achievements, and an individual’s self-efficacy in a particular domain can generalize to other domains. It is the overall self-confidence and sense of competence that individuals face environmental demands or new environments (Schwarzer et al., 1997), and it is also a personal resource (An et al., 2021). Previous studies have shown that self-efficacy can mediate between external environmental factors and individual status (Huang and Chen, 2012). Therefore, when facing the new working environment of remote work, it is worth further exploring whether external monitoring can affect individual employees through the mediating role of self-efficacy.

The mediating role of self-efficacy in the relationship between supervisors’ interactive monitoring and employee engagement and deviant behavior in remote workplaces.

In remote workplaces, because they are invisible, employees may worry that their leaders do not know their performance and doubt whether they will be fairly evaluated (Niehoff and Moorman, 1993). Supervisors interactively monitor their subordinates and obtain information by communicating with them. In this case, on the one hand, employees have a fair opportunity to introduce the details of their work progress (Mishra and Ghosh, 2020). On the other hand, employees can express their concerns and opinions, making them more likely to feel that they are being treated fairly (Wu and Wang, 2020). This sense of fairness from interactive monitoring helps to enhance employees’ self-efficacy (Fang, 2014). At the same time, interactive monitoring can help remote employees overcome and control environmental influences and feel capable during interactions (Khan et al., 2020). Additionally, in interactive monitoring, on the one hand, subordinates feel that their work is being observed by their superiors, which promotes their sense of competence. On the other hand, regular interactive discussions as a monitoring method provide subordinates with constructive problem-solving channels from their leaders, enhancing their ability to complete tasks (Mishra and Ghosh, 2020). In summary, supervisors’ interactive monitoring in remote workplaces serves as a work resource that helps to enhance employees’ self-efficacy, an individual psychological resource. Employees with high self-efficacy exhibit more positive states and fewer adverse behaviors (Kim and Beehr, 2017). Therefore, this study hypothesizes that self-efficacy can mediate between supervisors’ interactive monitoring and employee engagement and deviant behavior in remote workplaces.

The role of self-efficacy in work engagement has been well-validated, and research confirms that work-related self-efficacy is a characteristic of employees who maintain a high level of work engagement during remote work (Mäkikangas et al., 2022). Furthermore, studies have shown that psychological resources such as self-efficacy (Xanthopoulou et al., 2007) and psychological capital (Grover et al., 2018) mediate the positive effect of work resources on work engagement. Therefore, this study hypothesizes that self-efficacy mediates between supervisors’ interactive monitoring and employee work engagement in remote workplaces. Specifically, supervisors’ interactive monitoring in remote workplaces can provide resources such as interactive guidance and encouraging feedback to promote employees’ self-efficacy as a psychological resource (Mäkikangas et al., 2022). This further promotes employees’ work engagement in remote work. Therefore, this study proposes the following hypothesis:

*H3a*: Self-efficacy mediates the relationship between supervisors’ interactive monitoring and work engagement in remote workplaces. Specifically, supervisors’ interactive monitoring in remote workplaces enhances employees’ self-efficacy, further improving their work engagement.

Previous studies have confirmed that self-efficacy can reduce employees’ deviant behavior (Kim and Beehr, 2017; Iqbal et al., 2021). In remote work, supervisors’ interactive monitoring can provide employees with necessary resource support, stimulating their self-efficacy (Kim and Beehr, 2017). This further reduces employees’ engagement in behaviors that harm their self-evaluation, such as deviant behavior (Huck et al., 2017). Therefore, this study proposes the following hypothesis:

*H3b*: Self-efficacy mediates the relationship between supervisors’ interactive monitoring and deviant behavior in remote workplaces. Specifically, supervisors’ interactive monitoring in remote workplaces enhances employees’ self-efficacy, reducing their deviant behavior.

The mediating role of self-efficacy in the relationship between supervisors’ electronic monitoring and employee engagement and deviant behavior in remote workplaces.

As a form of observational monitoring, electronic monitoring in remote workplaces can make employees feel constantly supervised, leading to pressure to perform tasks in a required manner and reducing their self-efficacy (Huang and Chen, 2012). Unlike interactive monitoring, electronic monitoring collects information about employees’ performance without participation, reducing their confidence in completing tasks and assessing their performance. This is demonstrated in two ways: (1) subordinates do not have a say in matters related to their performance, weakening their control over outcomes such as performance evaluations; (2) the lack of attention and effort to investigate subordinates’ information deprives them of valuable growth opportunities and may reduce their sense of competence (Mishra and Ghosh, 2020). Specifically, ubiquitous electronic monitoring can reduce employees’ autonomy and sense of self-responsibility, making them unable to arrange and manage their own behavior freely (Niehoff and Moorman, 1993), lowering self-efficacy. The level of self-efficacy affects the degree of effort employees are willing to exert and the duration of their persistence when faced with obstacles. The higher employees’ self-efficacy, the more actively they will respond to obstacles, and vice versa (Yu and Du, 2023). Therefore, electronic monitoring reduces employees’ self-efficacy and further leads to reduced work effort (such as reduced job engagement) and adverse work behaviors (such as deviant behavior) in areas where electronic monitoring is “invisible” (Niehoff and Moorman, 1993). In summary, it is hypothesized that:

*H4a*: Self-efficacy mediates the relationship between supervisors’ electronic monitoring and work engagement in remote workplaces. Specifically, supervisors’ electronic monitoring in remote workplaces reduces employees’ self-efficacy, further reducing their work engagement.

*H4b*: Self-efficacy mediates the relationship between supervisors’ electronic monitoring and deviant behavior in remote workplaces. Specifically, supervisors’ electronic monitoring in remote workplaces reduces employees’ self-efficacy, further increasing their deviant behavior.

## Methods

### Sample and process

The remote work driven by COVID-19 has become the norm for many enterprises, and this study is not limited by factors such as industry and culture; the selection method for research subjects is to find 40 familiar friends and ask them to help find their acquaintances for a questionnaire filling. Referring to existing practices, to ensure the questionnaire’s validity, they must refrain from distributing it in their own company, and only one subject can be found in each enterprise (Luo et al., 2018). With the subjects’ permission, researchers directly contact them through email to collect and distribute the questionnaire. To ensure anonymity, researchers get the unique ID (The last four digits of the phone number + the last four digits of the ID number) and the subjects’ email addresses, but no other information (such as name).

This study collected questionnaire data in two periods to minimize the common method bias. Demographics, supervisor interactions, and electronic monitoring information in remote workplaces were collected in the first stage. This stage involved 40 acquaintances, each recommending 10 subjects, resulting in 400 questionnaires being distributed and 386 valid questionnaires being returned. After 2 months, self-efficacy, work engagement, and deviant behavior questionnaires were completed in the second stage. We distributed it to the 386 valid subjects from the first stage, returning 353 valid questionnaires. As the research context is remote work, all subjects must have had remote work experience. Questionnaires from subjects with no or minimal remote work experience were excluded, and finally, this study collected 299 valid questionnaires with a total effective recovery rate of 74.75%.

Regarding demographic information, there were 164 women, accounting for 54.8% of the total, and 135 men, accounting for 45.2%. The average age was 32.58 years. A total of 245 people had bachelor’s or higher degrees, accounting for 81.9%. The average number of working years in the current enterprise is 6.83 years. Regarding the nature of the enterprise, state-owned enterprises accounted for 54.5%, private enterprises accounted for 33.8%, foreign enterprises accounted for 0.3%, public institutions accounted for 5.4%, and others accounted for 6%.

### Measures

We adopted established scales in English to gather data, which were adapted through Back-translation (Richard, 1970) to ensure the validity of it for Chinese interviewees. In addition, we also invited research experts and corporate employees in related fields to conduct testing, and discuss any areas of disagreement until consensus was reached (Cheng et al., 2021). Except for demographic information, a five-point Likert response anchors ranging from 1(strongly disagree) to 5 (strongly agree) were applied. In particular, this study added a question after the demographic variables: “Have you ever experienced remote work?” to determine if the subject is in line with the research context of this study.

#### Interactive monitoring

We applied 5 items established by Liao and Chun (2016). In reference to the practices of other scholars, we added remote workplaces to all items (Zhao and Yang, 2020). A sample item is “In remote workplaces, my superior often arranges online meetings with me to discuss my work progress.” (Cronbach’s *α* = 0.899).

#### Electronic monitoring

We applied 4 items established by Holland et al. (2015). In reference to the practices of other scholars, we added remote workplaces to all items (Zhao and Yang, 2020). A sample item is “In remote work, my company uses relevant software to monitor our work (such as taking screenshots every 5 min, etc.).” (Cronbach’s α = 0.865).

#### Work engagement

We applied 3 items established by Schaufeli et al. (2019). In reference to the practices of other scholars, we added remote workplaces to all items (Zhao and Yang, 2020). A sample item is “In remote workplaces, I feel full of energy for my work.” (Cronbach’s *α* = 0.928).

#### Deviant behavior

We applied 12 items established by Stewart et al. (2009). In reference to the practices of other scholars, we added remote workplaces to all items (Zhao and Yang, 2020). A sample item is “In remote workplaces, I deliberately slow down my work speed.” (Cronbach’s *α* = 0.953).

#### Self-efficacy

We applied 10 items established by Schwarzer et al. (1997). A sample item is “I can face difficulties calmly because I believe in my ability to solve problems.” (Cronbach’s *α* = 0.922).

#### Control variables

By referring to existing studies, we selected gender, age, education background and tenures as control variables, which can influence employees’ work engagement and deviant behavior.

## Results

### Common method variance test

Although we carried out a two-wave data collection within 2 months in this study, and tried to dispel the misgivings of the respondents during the survey, yet, there might be a common method variance given that all the data came from respondents’ self-evaluation. Therefore, we employed Harman single-factor method (Harman, 1976) to test whether this variance exists. Thirty-four items from exploratory factor analysis (EFA) were utilized in the test. The results showed that only 6 factors’ eigenvalues exceeded 1 with the first factor referring to autonomy explained 31.41%, which was much lower than 50%. These results clearly demonstrates that common-method variance is not a serious problem in this study (Podsakoff et al., 2003; Malhotra et al., 2006).

### Validity test

Prior to examining the research hypotheses proposed in our model, a multi-level confirmatory factor analysis (CFA) widely adopted and validated by previous literature was carried out to confirm whether our study variables (interactive monitoring, electronic monitoring, self-efficacy, work engagement and deviant behavior) have good discriminant validity. Analysis with AMOS 23 showed that our five-factor model produced a reasonably good fit ( $\frac{\chi 2}{df}$ =1.874, TLI = 0.940, CFI = 0.946, RMSEA = 0.054), and fits better than alternative parsimonious models (Table 1). These results not only suggested that the latent constructs used in this study have acceptable discriminant validity, but also further certified that common-method variance is not a serious problem in our study.

**Table 1:** Results for confirmatory factor analysis.

Model	$\chi^{2}$	df	$\frac{\chi^{2}}{df}$	IFI	TLI	CFI	RMSEA
IM, EM, SE, WE, DB	946.284	505	1.874	0.946	0.940	0.946	0.054
IM + EM, SE, WE, DB	1791.551	509	3.520	0.844	0.827	0.843	0.092
IM + SE, EM, WE, DB	1689.563	509	3.319	0.856	0.841	0.856	0.088
IM, EM + SE, WE, DB	1718.214	509	3.376	0.853	0.837	0.852	0.089
IM + SE + WE, EM, DB	2263.293	512	4.420	0.787	0.765	0.786	0.107
IM, EM + SE + DB, WE	2933.301	512	5.729	0.706	0.675	0.704	0.126
IM + SE + WE, EM + DB	2815.246	514	5.477	0.720	0.693	0.718	0.123
EM + SE + DB, IM + WE	3549.421	514	6.905	0.631	0.595	0.629	0.141

*N* = 299; IM, interactive monitoring; EM, electronic monitoring; SE, self-efficacy; WE, work engagement; DB, deviant behavior.

### Descriptive statistics

Table 2 presents the means, standard deviations, and correlations of the study variables, which were run by SPSS 23. The results suggest a positive correlation between interactive monitoring and self-efficacy (*r* = 0.275, *p* < 0.01), a positive correlation between interactive monitoring and work engagement (*r* = 0.434, *p* < 0.01), a negative correlation between electronic monitoring and self-efficacy (*r* = −0.174, *p* < 0.01), a positive correlation between electronic monitoring and deviant behavior (*r* = 0.462, *p* < 0.01), a positive correlation between self-efficacy and work engagement (*r* = 0.490, *p* < 0.01), a negative correlation between self-efficacy and deviant behavior (*r* = −0.244, *p* < 0.01), which provides preliminary evidence for subsequent hypothesis testing.

**Table 2:** Means, standard deviations and correlation coefficients of variables.

Variables	*M*	SSD	1 Interactive motoring	2 Electronic motoring	3 Self-efficacy	4 Work engagement	5 Deviant behavior	6 Gender	7 Age	8 Education background
1	3.319	0.934	(0.899)							
2	1.951	0.821	0.007	(0.865)						
3	3.609	0.575	0.275^**^	−0.174^**^	(0.922)					
4	3.589	0.820	0.434^**^	0.006	0.490^**^	(0.928)				
5	1.813	0.716	−0.101	0.462^**^	−0.244^**^	−0.214^**^	(0.953)			
6	1.550	0.498	−0.092	−0.071	−0.055	0.038	−0.267^**^			
7	32.580	7.240	−0.076	−0.067	0.069	0.108	−0.075	−0.129^*^		
8	2.920	0.678	−0.028	−0.115^*^	0.047	−0.086	−0.070	0.136^*^	−0.226^**^	
Tenure	6.830	5.513	−0.048	0.051	0.063	0.110	0.007	−0.165^**^	0.624^**^	−0.180^**^

*N* = 299, ^**^p < 0.010, ^*^p < 0.050.

### Hypothesis tests

We tested the hypotheses H1a, H1b, H2a, and H2b, meanwhile preliminarily examined the mediating effect via hierarchical regression using SPSS 23. The results are presented in Table 3. Further, referring to the suggestion of Edwards and Lambert (2007), we applied Bootstrap (5,000 times) to verify the mediating effect (H3a, H3b, H4a, and H4b).

**Table 3:** Results for hierarchical regression analysis.

Independent variables	Dependent variables						
	Self-efficacy	Work engagement			Deviant behavior		
	Model 1	Model 2	Model 3	Model 4	Model 5	Model 6	Model 7
Control							
Gender	−0.039	0.185^*^	0.153	0.208^**^	−0.374^**^	−0.408^**^	−0.381^**^
Education background	0.053	−0.063	−0.128^*^	−0.094	−0.010	−0.043	0.000
Age	0.004	0.011	0.003	0.008	−0.008	−0.014	−0.007
Tenure	0.006	0.012	0.009	0.008	−0.002	0.007	−0.001
Dependent variables							
Interactive monitoring	0.174^**^	0.398^**^		0.296^**^	−0.104^**^		−0.070
Electronic monitoring	−0.119^**^	0.007		0.078	0.382^**^		0.359^**^
Mediator							
Self-efficacy			0.706^**^	0.589^**^		−0.314^**^	−0.196^**^
F	6.598	14.104	21.074	24.926	20.084	10.433	19.015
ΔF	17.739^**^	38.064^**^	96.382^**^	69.890^**^	42.031^**^	21.668^**^	9.209^**^
*R* ^2^	0.119	0.225	0.265	0.375	0.292	0.151	0.314
$\Delta R^{2}$	0.107^**^	0.202^**^	0.242^**^	0.150^**^	0.204^**^	0.063^**^	0.022^**^

*N* = 299, ^**^P < 0.010, ^*^P < 0.05.

Hypothesis 1a proposes that interactive monitoring is positively associated with work engagement. As anticipated, results from Table 3 reports that Hypothesis 1a is supported ( β= 0.398, *p* < 0.01, Model 2).

Hypothesis 1b proposes that interactive monitoring is negatively associated with deviant behavior. As predicted, results from Table 3 reports that Hypothesis 1b is supported( β= –0.104, *p* < 0.01, Model 5).

Hypothesis 2a proposes that electronic monitoring is negatively associated with work engagement. The results in Table 3 reports that H2a is not supported( β= 0.007, *n.s.*, Model 2).

Hypothesis 2b proposes that electronic monitoring is positively associated with deviant behavior. As anticipated, results from Table 3 reports that Hypothesis 2b is supported ( β= 0.382, *p* < 0.01, Model 5).

Hypothesis 3a proposes that interactive monitoring indirectly affects work engagement via self-efficacy. To compare Model 3 and Model 4, we add monitoring in Model 4 based on Model 3, which still suggests a positive association between self-efficacy and work engagement ( β= 0.598, *p* < 0.01, Model4). Although the effect turns weaker, it’s still significant. Furthermore, to compare Model 2 and Model 4, we add self-efficacy in Model 4 based on Model 2, which still suggests a positive association between interactive monitoring and work engagement( β= 0.296, *p* < 0.01, Model 4). The effect is still significant indicating a partial mediation. H3a is supported.

Referring to the suggestions of Edwards and Lambert (2007), Bootstrap (5,000 times) is applied in this study to further verify the indirect effect of self-efficacy in the model. The results are shown in Table 4, demonstrating that this indirect effect as hypothesized is pronounced (estimate = 0.097, 95%CI =0.044, 0.168), which confirms that interactive monitoring indicates a significant mediating effect on work engagement through self-efficacy. And the direct estimate is significant (estimate = 0.284, 95%CI = 0.199,0.369), so self-efficacy partially mediates the relationship between interactive monitoring and work engagement. The above results provide support for hypothesis 3a.

**Table 4:** Bootstrap test results for direct effect and indirect effect of self-efficacy in interaction supervisor and work engagement.

Path	Estimates	SE	95%CI	
			Lower	Upper
Total	0.381^**^	0.046	0.290	0.471
Indirect Direct	0.097^**^ 0.284^**^	0.032 0.043	0.044 0.199	0.168 0.369

*N* = 299, ^**^P < 0.010, ^*^P < 0.050.

Hypothesis 3b proposes that interactive monitoring indirectly affects deviant behavior via self-efficacy. To compare Model 6 and Model 7, we add monitoring in Model 7 based on Model 6, which still suggests negative association between self-efficacy and deviant behavior( β= –0.196, *p* < 0.01, Model7). Although the effect turns weaker, it’s still significant. Furthermore, to compare Model 5 and Model 7, we add self-efficacy in Model 7 based on Model 5, which suggests the negative association between interactive monitoring and deviant behavior is not significant( β= –0.070, *n.s.*, Model 7). So the mediating role of self-efficacy is not significant.

Hypothesis 4a proposes that electronic monitoring indirectly affects work engagement via self-efficacy. Because the direct effect between electronic monitoring and work engagement is not significant (Model 2), it’s unnecessary to confirm the indirect effect. H4a is not supported.

Hypothesis 4a proposes that electronic monitoring indirectly affects deviant behavior via self-efficacy. To compare Model 6 and Model 7, we add monitoring in Model 7 based on Model 6, which still suggests a negative association between self-efficacy and deviant behavior ( β= –0.196, *p* < 0.01, Model 7). Although the effect turns weaker, it’s still significant. Furthermore, to compare Model 5 and Model 7, we add self-efficacy in Model 7 based on Model 5, which still suggests a positive association between electronic monitoring and deviant behavior( β= 0.359, *p* < 0.01, Model 7). The effect is still significant indicating a partial mediation. H4b is supported.

Referring to the suggestions of Edwards and Lambert (2007), Bootstrap (5,000 times) is applied in this study to further verify the indirect effect of self-efficacy in the model. The results are shown in Table 5, demonstrating that this indirect effect as hypothesized is pronounced (estimate = 0.026, 95%CI = 0.006, 0.054), which confirms that electronic monitoring indicates a significant mediating effect on deviant behavior through self-efficacy. And the direct estimate is significant (estimate = 0.377, 95%CI = 0.289, 0.465), so self-efficacy partially mediates the relationship between electronic monitoring and deviant behavior. The above results provide support for hypothesis 4b.

**Table 5:** Bootstrap test results for direct effect and indirect effect of self-efficacy in monitor supervisor and deviant behavior.

Path	Estimates	SE	95%CI	
			Lower	Upper
Total	0.403^**^	0.045	0.314	0.491
Indirect Direct	0.026^**^ 0.377^**^	0.013 0.045	0.006 0.289	0.054 0.465

*N* = 299, ^**^P < 0.010, ^*^P < 0.050.

**Figure 2:**
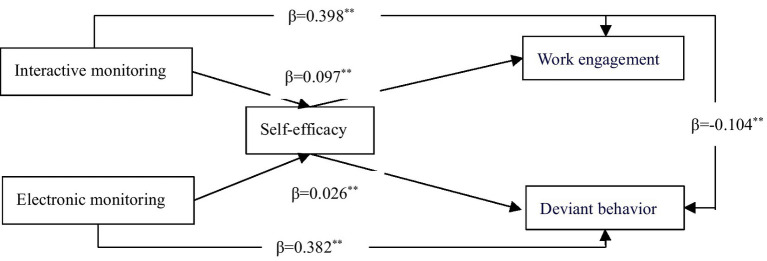
Final validated research model.

After the hypothesis tests, the final validated research model is presented in Figure 2.

## Discussion

### Conclusion

This study focuses on supervisor monitoring in remote workplaces based on the Job Demands-Resources (JD-R) model. It examines the impact of different monitoring methods on remote employees’ work engagement and deviance via the mediating effect of self-efficacy. Based on 299 valid data collected in two periods, the following research findings are as follows.

The interactive monitoring in remote workplaces has a significant positive impact on employees’ work engagement (H1a is supported), and a significant negative impact on employees’ deviance (H1b is supported). Self-efficacy plays a mediating role between interactive monitoring and work engagement (H3a is supported), but it does not mediate the relationship between interactive monitoring and deviant behavior (H3b is not supported). Electronic monitoring by superiors in remote workplaces has a significant positive impact on employees’ deviant behavior (H2b is supported), but it does not have a significant impact on employees’ work engagement (H2a and H4a are not supported). Self-efficacy mediates the relationship between electronic monitoring and deviant behavior (H4b is supported).

Although some hypotheses in this study were not supported, the results are consistent with existing research. Schaufeli and Bakker, (2004) found that job resources can increase work engagement but not necessarily reduce deviance, while job demands can increase deviance but not necessarily reduce work engagement. Interactive monitoring by superiors in remote workplaces is a type of job resource that enhances work engagement by promoting personal resources such as self-efficacy, but it does not reduce deviance. Electronic monitoring in remote workplaces is a type of job demand that increases employee deviance by depleting personal resources such as self-efficacy, but it does not reduce work engagement.

### Theoretical implications

First and foremost, this study contributes to the research on leadership and employee behavior in remote workplaces. With remote work becoming increasingly popular and expected to gain further prevalence, there is a need for more research on how to improve employee engagement and reduce deviant behavior (Huo et al., 2022). This study enhances the understanding of relevant research by examining the impact of different monitoring approaches by superiors on improving employee engagement and reducing deviant behavior in remote workplaces.

Secondly, this study expands the research context of supervisor monitoring. The current research on the effect of supervisors’ monitoring is mainly based on traditional offline workplaces (Zhou, 2003; Liao and Chun, 2016; Son et al., 2017; Mishra and Ghosh, 2020). However, by limiting the research context to remote workplaces, including subjects with remote work experience within the scope of valid data, and specifying remote work scenarios in the survey questions, this study provides strong evidence for the effectiveness of supervisor monitoring in remote workplaces.

Once again, this study enhances the theoretical perspective of the influence mechanism of supervisor monitoring. The current research on the effect of supervisors’ monitoring mainly depended on the perspectives of social exchange theory (Liao and Chun, 2016; Son et al., 2017) and self-determination theory (Zhou, 2003; Mishra and Ghosh, 2020). However, by distinguishing the different effects of interactive and electronic monitoring from supervisors through the lens of job demands-resources, this study highlights that supervisor monitoring in remote workplaces can serve as both a job resource and a job demand, depending on the specific monitoring approach. This further complements the research on the differentiated effects of interactive and observational monitoring by superiors. Additionally, it echoes the varying degrees of influence of job resources and job demands on positive and negative outcomes, as suggested by Schaufeli and Bakker, (2004), where job resources increase work engagement but do not necessarily reduce deviance, while job demands increase deviance but do not necessarily reduce work engagement.

### Practical implications

This study focuses on the effectiveness of supervisor monitoring in remote workplaces, which aligns with current management practice requirements. The research findings can provide the following insights to remote work managers.

When managing remote work, supervisors can adopt more interactive monitoring, such as regularly communicating and exchanging with employees, holding online meetings periodically, listening to employees’ self-reported work performance and problems, and providing timely interactive feedback and assistance to address employees’ deficiencies in remote workplaces. This approach can help remote employees increase their work engagement and, to some extent, prevent a decrease in organizational identity caused by organizational isolation in remote work (Xiao, 2019).

It is recommended that organizations reduce the use of electronic monitoring in remote workplaces, as this can further increase employees’ work stress and sense of intrusion in remote workplaces, thereby enhancing their deviant behavior and hindering work efficiency.

Self-efficacy is a positive individual resource for remote work employees. Organizations should focus more on developing employees’ psychological capital and consider enhancing their self-efficacy as an essential construction means. This can increase remote work employees’ engagement and reduce their deviant behavior.

### Limitations and directions of future research

First, although we carried out a two-wave data collection within 2 months in this study, yet there might still be a common method variance given that all the data came from respondents’ self-report. Therefore, future researchers are strongly encouraged to adopt a multi-stage and a multi-source (e.g., employees, leaders, team members, etc.) questionnaire design, in order to obtain more scientific and effective research results.

Second, many studies have confirmed that employees’ work engagement fluctuates significantly daily, so it is recommended to consider using a diary method to measure relevant variables to obtain more effective research data.

Third, this study just tests work engagement and deviant behavior as the results of supervisor monitoring in remote workplaces, not test performance, which is most important to organizations. So, in future research, it is necessary to verify the impact of different leadership supervision methods on employee work performance in remote work situations.

Fourth, regarding the research context, this study only treated remote work as an overall variable measurement context. Future research can make more detailed distinctions. On the one hand, a comparative analysis can examine the effectiveness of supervisor monitoring in remote workplaces and traditional face-to-face workplaces for the same participants. On the other hand, the intensity of remote work can be controlled during the study to enable more effective analysis.

Regarding boundary conditions, as this study primarily aims to discuss the effectiveness of remote workplaces, moderators were not selected for boundary condition verification. Future research can explore factors such as different individual characteristics (e.g., proactiveness, responsibility) or work characteristics (e.g., result-oriented work) for further exploration.

## Data availability statement

The raw data supporting the conclusions of this article will be made available by the authors, without undue reservation.

## Ethics statement

Ethical review and approval was not required for the study on human participants in accordance with the local legislation and institutional requirements. Written informed consent from the patients/participants or patients/participants legal guardian/next of kin was not required to participate in this study in accordance with the national legislation and the institutional requirements.

## Author contributions

SL: Conceptualization, Data curation, Formal analysis, Funding acquisition, Investigation, Methodology, Project administration, Resources, Software, Supervision, Validation, Visualization, Writing – original draft, Writing – review & editing. YW: Funding acquisition, Methodology, Supervision, Writing – review & editing.
